# Hypoxia Adaptations in the Grey Wolf (*Canis lupus chanco*) from Qinghai-Tibet Plateau

**DOI:** 10.1371/journal.pgen.1004466

**Published:** 2014-07-31

**Authors:** Wenping Zhang, Zhenxin Fan, Eunjung Han, Rong Hou, Liang Zhang, Marco Galaverni, Jie Huang, Hong Liu, Pedro Silva, Peng Li, John P. Pollinger, Lianming Du, XiuyYue Zhang, Bisong Yue, Robert K. Wayne, Zhihe Zhang

**Affiliations:** 1 Sichuan Key Laboratory of Conservation Biology on Endangered Wildlife, Chengdu research base of giant panda breeding, Chengdu, Sichuan Province, P. R. China; 2 Department of Ecology and Evolutionary Biology, UCLA, Los Angeles, California, United States of America; 3 Key Laboratory of Bioresources and Ecoenvironment (Ministry of Education), College of Life Sciences, Sichuan University, Chengdu, People's Republic of China; 4 ISPRA, Ozzano dell'Emilia, Italy; 5 Sichuan Key Laboratory of Conservation Biology on Endangered Wildlife, College of Life Sciences, Sichuan University, Chengdu, P. R. China; 6 CIBIO-UP, University of Porto, Vairão, Portugal; University of Washington, United States of America

## Abstract

The Tibetan grey wolf (*Canis lupus chanco*) occupies habitats on the Qinghai-Tibet Plateau, a high altitude (>3000 m) environment where low oxygen tension exerts unique selection pressure on individuals to adapt to hypoxic conditions. To identify genes involved in hypoxia adaptation, we generated complete genome sequences of nine Chinese wolves from high and low altitude populations at an average coverage of 25× coverage. We found that, beginning about 55,000 years ago, the highland Tibetan grey wolf suffered a more substantial population decline than lowland wolves. Positively selected hypoxia-related genes in highland wolves are enriched in the HIF signaling pathway (*P* = 1.57E-6), ATP binding (*P* = 5.62E-5), and response to an oxygen-containing compound (*P*≤5.30E-4). Of these positively selected hypoxia-related genes, three genes (*EPAS1*, *ANGPT1*, and *RYR2*) had at least one specific fixed non-synonymous SNP in highland wolves based on the nine genome data. Our re-sequencing studies on a large panel of individuals showed a frequency difference greater than 58% between highland and lowland wolves for these specific fixed non-synonymous SNPs and a high degree of LD surrounding the three genes, which imply strong selection. Past studies have shown that *EPAS1* and *ANGPT1* are important in the response to hypoxic stress, and *RYR2* is involved in heart function. These three genes also exhibited significant signals of natural selection in high altitude human populations, which suggest similar evolutionary constraints on natural selection in wolves and humans of the Qinghai-Tibet Plateau.

## Author Summary

Understanding the genetic mechanisms that allow some individuals to live at high altitudes under hypoxic conditions can provide insight into the evolutionary constraints of adaptation to extreme conditions and the development of hypoxia-related disease in humans. The Tibetan grey wolf (*Canis lupus chanco*) has long existed on the Qinghai-Tibet Plateau, where low oxygen tension exerts unique selection pressure on individuals. Comparing the complete genome sequences of 4 grey wolves from high altitude and 5 from low altitude, we identify three candidate genes for high-altitude adaptation (*EPAS1*, *ANGPT1*, and *RYR2*) that show strong signals of selection. The three genes potentially enhance function under hypoxic conditions by increasing oxygen delivery (*EPAS1* and *ANGPT1*) and heart (*RYR2*) function. These genes also appear under selection in high altitude human populations, which suggesting there may be limited pathways for adapting to high altitude existence.

## Introduction

Species inhabiting the Qinghai-Tibet Plateau exist in low oxygen tension environments and must adapt to low oxygen tension [1]. Documenting the genetic mechanisms for adaptation to hypoxia can provide insights into the process of evolution under extreme conditions and hypoxia-related disease in humans. Compared with their lowland counterparts, Tibetan human populations show unique adaptations, such as low hypoxic pulmonary vasoconstrictor response, high levels of blood oxygen saturation, and low hemoglobin (Hb) levels [1]–[2]. The genetic basis for some of these phenotypes has been identified and hypoxia-related genes (such as *EPAS1*, *EGLN1*) have experienced strong selection in Tibetans [3]–[10]. Past studies of complete genomes have not found these genes to be under selection in other highland vertebrates, such as deer mice [11], the yak [12], the ground tit [13], and Tibetan antelope [14]. However, these studies have used a comparative genomic approach involving the analysis of genome sequences from a variety of divergent species. A potentially more powerful approach utilizes complete genome sequences from populations of the same species, conditional on the knowledge of their demographic history and gene flow.

The grey wolf, *Canis lupus*, is the most widely distributed terrestrial mammal with a geographic range spanning latitudinal and altitudinal gradients and including as many as 32 sub-species [15]. Rates of gene flow among grey wolf populations are high [16], reflecting large average dispersal distances [17]. Nonetheless, significant genetic differences among populations have been identified that correlate with environmental variation, suggesting a process of habitat selection that is based on natal conditioning [18]. The Tibetan grey wolf (*C. lupus chanco*) is a relatively large form having a more wooly coat [19]–[20] that occupies habitats on the Qinghai-Tibet Plateau, implying local adaptation to low oxygen tension [12]. The genetic basis of such adaptation, however, remains unknown. Here we compare the whole genomes of low- and high- altitude populations of wolves from China in order to explore adaptation to hypoxia in the Qinghai-Tibet Plateau wolf population.

## Results

### Genome data

**Figure 1:**
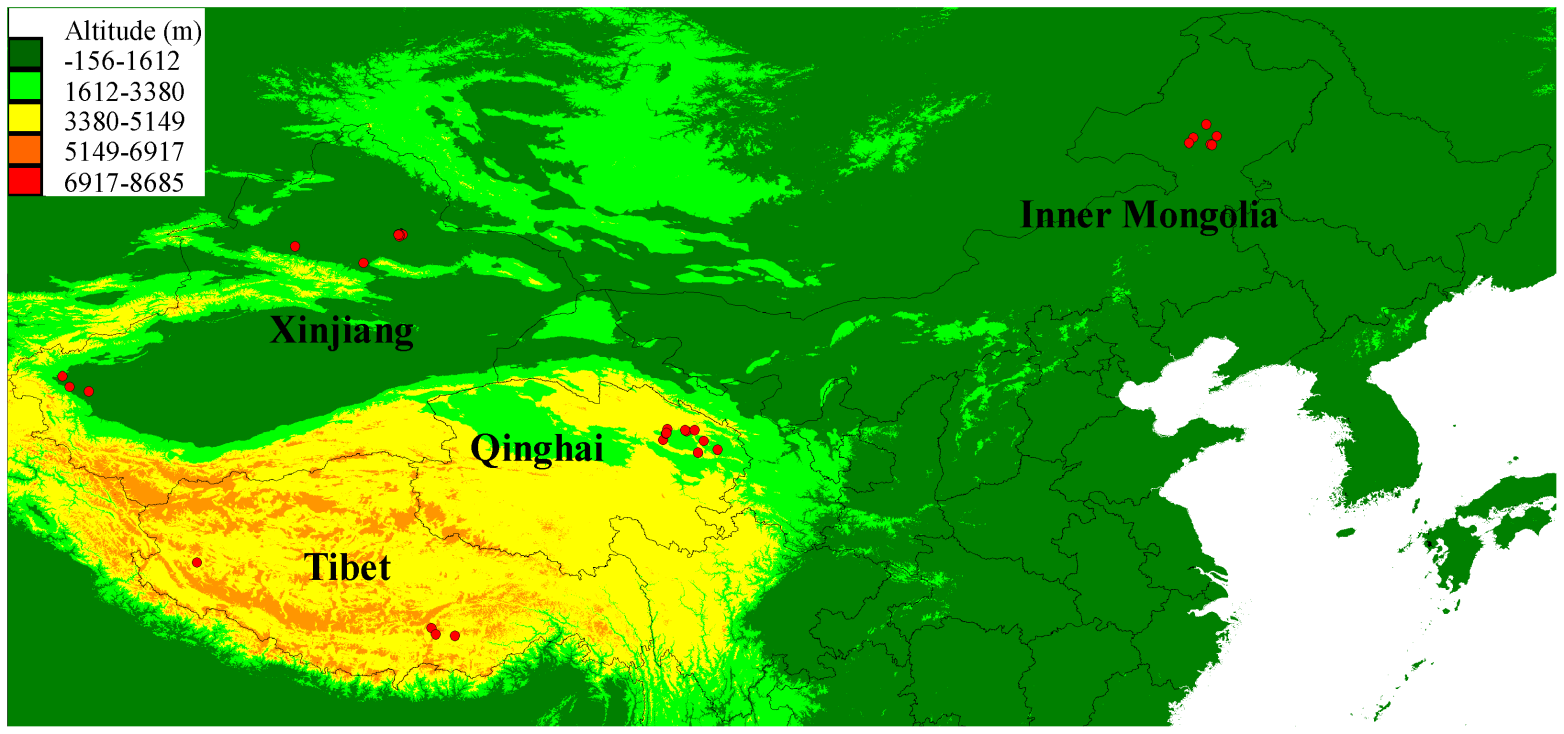
Geographical distribution of the sampled 35 Chinese wolves in this study. The average altitudes are also shown on the map.

Eight wolves that represented four distinct populations from lowland (Xinjiang and Inner Mongolia) and highland (Tibet and Qinghai) (Fig. 1 and S2; Table S1) were selected for genome sequencing on an Illumina HiSeq 2000 platform. The short reads from an additional wolf from one locality in Inner Mongolia used on a previous study (RKWL [21]) were also included.

**Figure 2:**
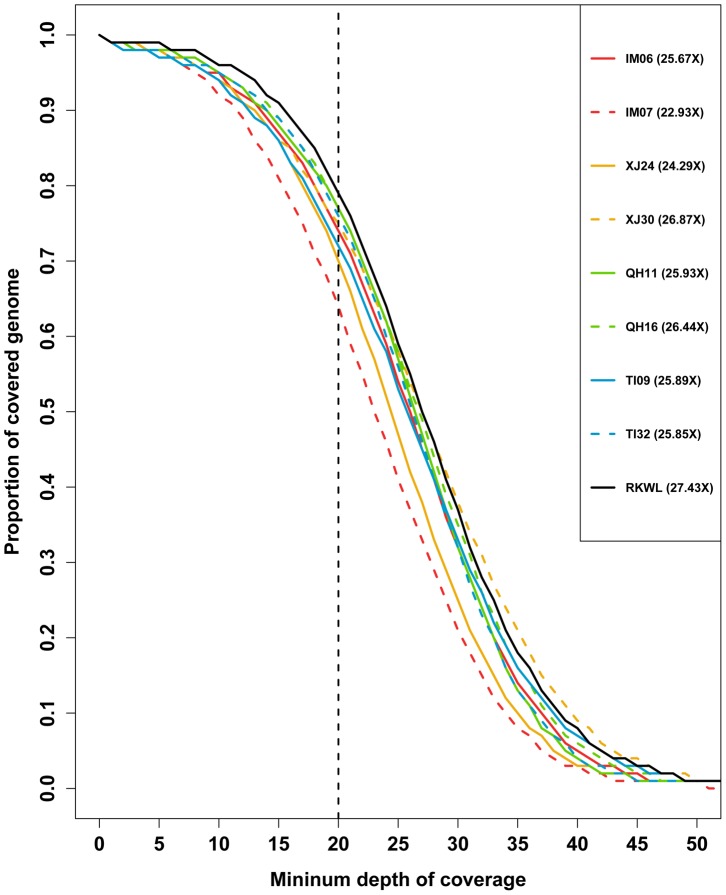
Depth of coverage of the nine Chinese wolf genomes. The proportion of the genome exceeding a given minimum depth of coverage is shown. The numbers in legend are the mean genome wide coverage.

PCR duplicates were detected and marked in Picard (http://picard.sourceforge.net). From 6.69% to 15.96% of reads in each sample were PCR duplicates and all of the PCR duplicates were excluded from downstream analyses. Then, after removing adapters and low quality reads (quality value of < = 5 for > = 50% of reads), we generated 5.9 billion 100-bp reads in total (from 686 to 763 million reads per sample), which covered 535.84 Gb bases (from 61.3 Gb to 68.69 Gb per sample; Table S2). All the alignments were done in Bowtie2, and each individual had more than a 98% alignment rate (Table S2). After running the genotyping pipeline, the total coverage of these nine Chinese wolves was 231.3-fold and every sample had more than 20-fold effective genome-wide mean coverage (Fig. 2; Table S2). In order to further control the data quality and remove false positives, we applied genome (GF2) and sample filters (SF) to obtain a final SNP dataset for each Chinese wolf. All the samples had more than 1.3 billion total useable sites, which covered more than 60% of the reference genome (61.97%∼63.13%; Table S3). Slightly more than 1% of the total sites were filtered out in each individual by excluding the CpG sites (Table S4). The number of SNPs was very similar among individuals (about 3 million, from 2,925,783 to 3,482,449). However, the two wolves from Tibet (2,925,783 and 3,010,114) had relative fewer SNPs (Table S3). To quantify genome-wide heterozygosity, for each wolf we calculated the ratio of passed-filter heterozygous genotype calls against all passed-filters sites (GF2 and SF; Table S3). The autosomal heterozygosity was the highest in two Xinjiang wolves (0.001597 and 0.001632) and the lowest in two Tibet wolves (0.000705 and 0.000862), which is only about half that in Xinjiang wolves (Table S3). Highland wolves had lower autosomal heterozygosity than lowland wolves (*P* = 0.03, one-way ANOVA).

Based on the SNPs from each sample, we created a merged SNPs dataset. Across these nine wolves, we found 7,509,614 SNPs in the merged dataset. After the removal of sites that were missing in one or more the samples, 6,645,354 SNPs remained in the merged SNP dataset (Table S3 and S4).

### Population structure

A dataset of 266,299 pruned SNPs was obtained from the 7,509,614 SNPs of the nine Chinese wolves after removing SNPs with high pairwise genotypic association.

**Figure 3:**
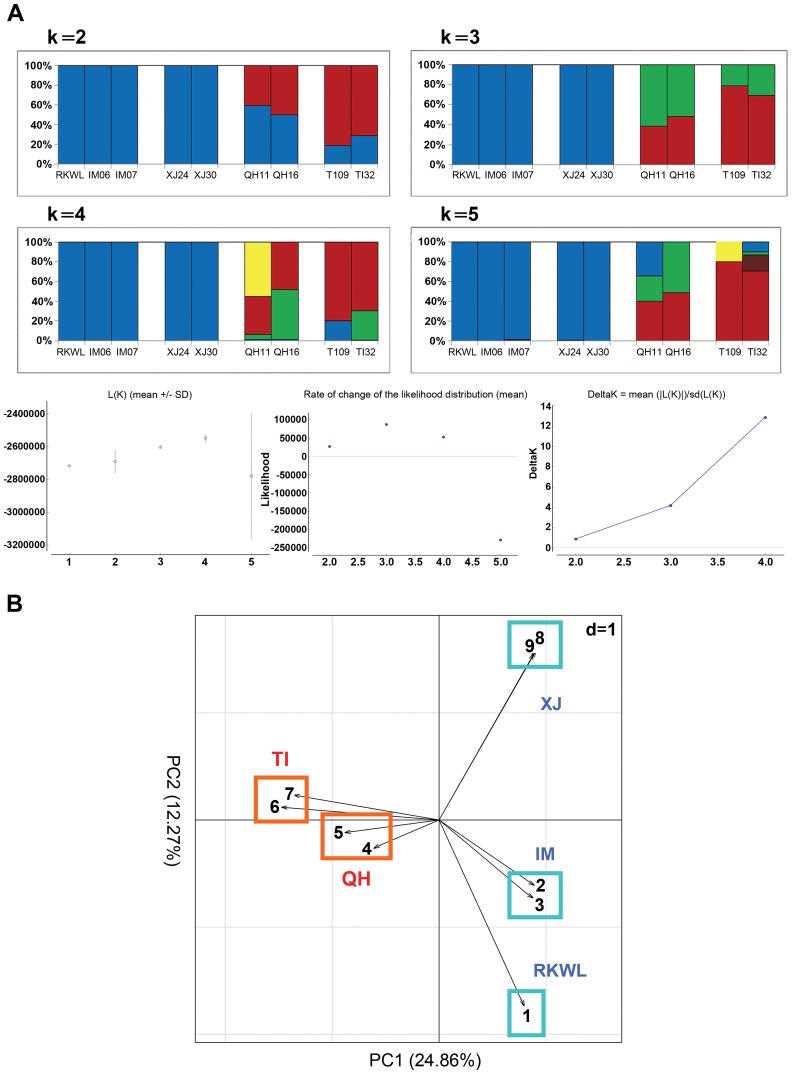
Population structure from genome data (excluding highly linked SNPs with r^2^>0.2). A: Structure assignments based on complete genome data from nine Chinese wolves. B: Principle component analysis of complete genome data from nine Chinese wolves. Xinjiang, XJ: XJ24, XJ30; Inner Mongolia, IM: IM06, IM07; Tibet, TI: TI09, TI32; Qinghai, QH: QH11, QH16. See table S1 and the text for abbreviations and localities.

The genome wide STRUCTURE results showed that the five lowland wolves (IM06, IM07, RKWL, XJ24, and XJ30) grouped with each other whereas the four highland wolves (TI09, TI32, QH11, and QH16) were in another group from K = 2 to K = 5 (Fig. 3). The likelihood values was highest for K = 3 or K = 4 (Fig. 3). Qinghai appears as a transitional zone between Tibet and lowland (Xingjiang and Inner Mongolia) populations (Fig. 1), which was consistent with the STRUCTURE result because at K = 2, the Qinghai individuals were intermediate between Tibet and lowland wolves (Fig. 3A).

The PCA exhibited a clearer picture of the groupings. PC1 explained 24.86% while PC2 explained 12.27% of the overall variation (Fig. 3B). The four highland wolves clustered together, while the five lowland wolves were significantly separated from the highland wolves along PC1. Unlike the STUCTURE results, PC2 strongly separated Xinjiang wolves and Inner Mongolian wolves from each other, but the four highland wolves were still clustered together. These results were also consistent with the geographic distribution of the samples (Fig. 1).

### Demography

**Figure 4:**
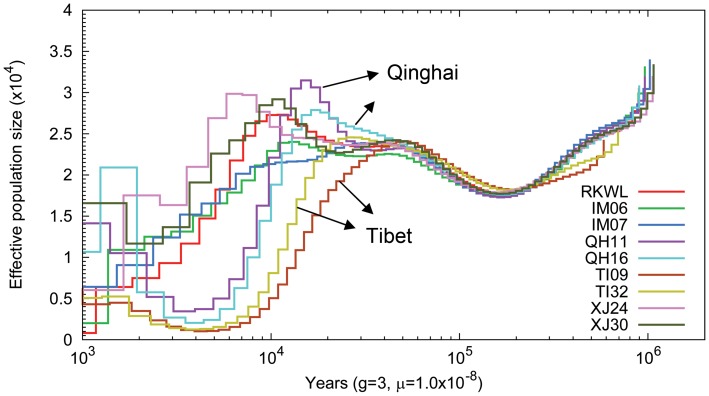
Pairwise sequential Markovian coalescent (PSMC) analysis of nine Chinese wolf genomes reflecting the genomic distribution of heterozygous sites. Time scale on the x-axis is calculated assuming a mutation rate of 1×10^−8^ per generation and generation time equal to 3.

The pairwise sequentially Markovian coalescent model (PSMC [22]) revealed that the nine Chinese wolves exhibited similar demographic trajectories until about 80,000 years ago (Fig. 4). Thereafter, all populations except Tibet experienced some population growth or stagnation until about 24,000 years ago. The growth phase occurred around the Greatest Lake Period (30,000–40,000 years ago), during which the forested habitats appropriate for wolves increased [23]. In contrast, the two Tibetan wolves experienced a continuous population decline beginning 55,000 years ago. However, all wolf populations appeared to decline beginning at the last glacial maximum (21,000–17,000 years ago), when the expansion of glaciers in the northern hemisphere likely decreased the extent of the habitat suitable for wolves. However, PSMC loses resolution for dates earlier than about 20,000 year ago because of the lack of recombination events [21]–[22], so the timing of this event is less certain.

### Genome-wide selection and gene enrichment

**Figure 5:**
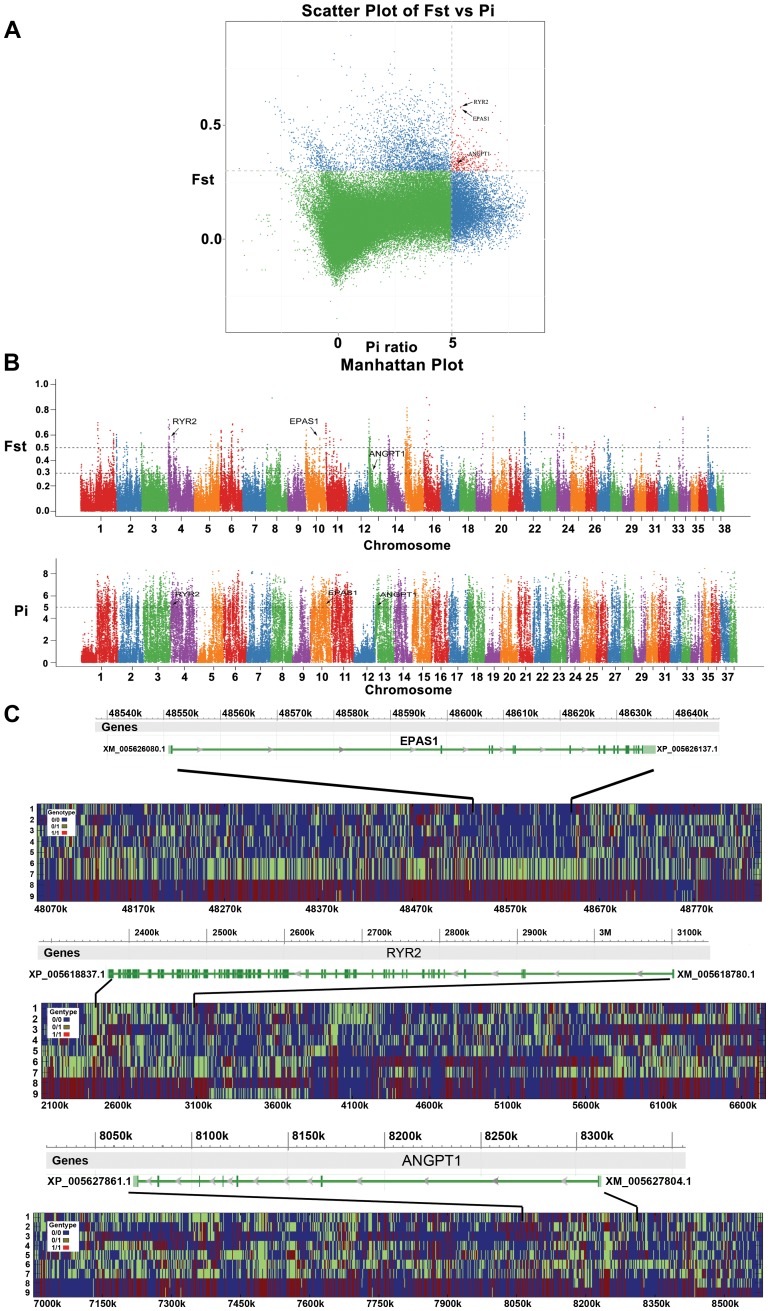
Genomic regions with strong selective sweep signals in Tibet wolves. The three hypoxia-related genes (*EPAS1*, *RYR2*, and *ANGPT1*) which each include at least one fixed non-synonymous SNP in highland wolves are highlighted. (A) Distribution of *θ*_π_ ratios (*θ* ^high^_π_/*θ* ^low^_π_) and F_ST_ values calculated in 100-kb sliding windows in 20-kb steps. Data points in red (corresponding to the 5% empirical *θ*_π_ ratio distribution, where *θ*_π_ ratio is 5.311, and the 5% empirical F_ST_ distribution, where F_ST_ is 0.259) are regions under selection in highland wolves. (B) Genome-wide distribution of F_ST_ and Δπ along autosomes 1–38 (chromosomes are separated by color). Each dot represents 100 Kb genome regions. A dashed horizontal line indicates the top 5% level (F_ST_>0.259; Pi>5.311) used for extracting outliers, where another dashed horizontal line F_ST_>0.489 shows the top 1% level. (C) The genotypes observed in 9 full-genome data around *EPAS1*, *RYR2*, and *ANGPT1*. Every plot includes a gene region (top) and genotypes around the region (below). The y-axis denotes individuals: 1—RKWL [21], 2-IM06, 3-IM07, 4-XJ24, 5-XJ30, 6-QH11, 7-QH16, 8-TI09, and 9-TI32. See table S1 and the text for abbreviations and localities. The x-axis denotes the locations on genome.

The genomic signatures of positive selection in highland wolves were evaluated using two metrics: F_ST_ and Δπ (Fig. 5). Using these metrics, we identified 902 outlier regions which included 1548 genes potentially under selection. The 1548 genes included 229 significant GO (Gene Ontology) terms (Table S6) and several significant enrichment categories included genes involved in response to stimulus (Table S6). Additionally, we defined an *a priori* candidate list of 1351 hypoxia-related genes (Table S5).

Of these 1548 putatively selected genes, 84 were potentially related to hypoxia (Table S5). The GO enrichment (Table S7) of the 84 hypoxia-related genes showed strong enrichment for the HIF (Hypoxia-Inducible Factors) signaling pathway (KEGG:04066, 6 genes), ATP binding (GO:0005524, 14 genes), and response to oxygen-containing compound (GO:1901700, 7 genes; GO:1901701, 5 genes).

### SNPs in coding regions

**Figure 6:**
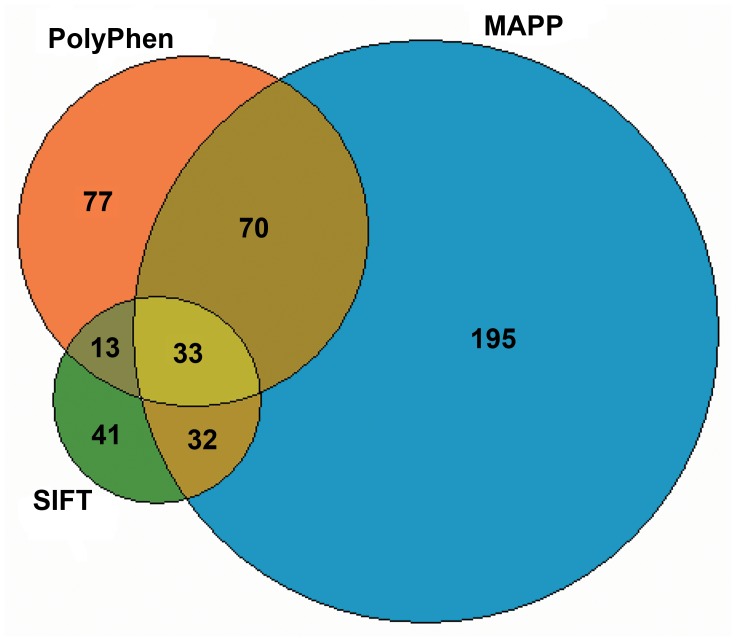
The number of nonsynonymous SNPs that might affect protein function based on SIFT [24], MAPP [25], and PolyPhen2 [26]. These SNPs genotypes differ at the 5% level between highland and lowland wolves.

A total of 2598 SNPs in coding regions were found with a significant difference at the 5% level between genotypes of highland and lowland wolves. Of these 2598 SNPs, 893 were non-synonymous in 683 genes ranging from 1 to 12 per gene. Fifty-two of these SNPs were from 43 hypoxia-related genes with 1 to 4 SNPs per gene. Finally, of the 893 non-synonymous SNPs, a relevant change in protein function was suggested for 119 SNPs with SIFT [24], 330 SNPs with MAPP [25], and 193 SNPs with PolyPhen2 [26] (Fig. 6). The three methods had in common 33 SNPs from 32 genes (Fig. 6). However, only one of these was a hypoxia-related gene (*RYR2*; Fig. 5).

### Re-sequencing

In total, 17210 bp of DNA sequences from 17 genes were re-sequenced in 35 wolves and 108 SNPs were found among them (Table S8). Of the 108 SNPs, 18 SNPs were not found in the whole genome data, 64 were located in introns, 34 in exons (14 synonymous and 20 nonsynonymous), and 10 in UTRs (Table S8).

**Figure 7:**
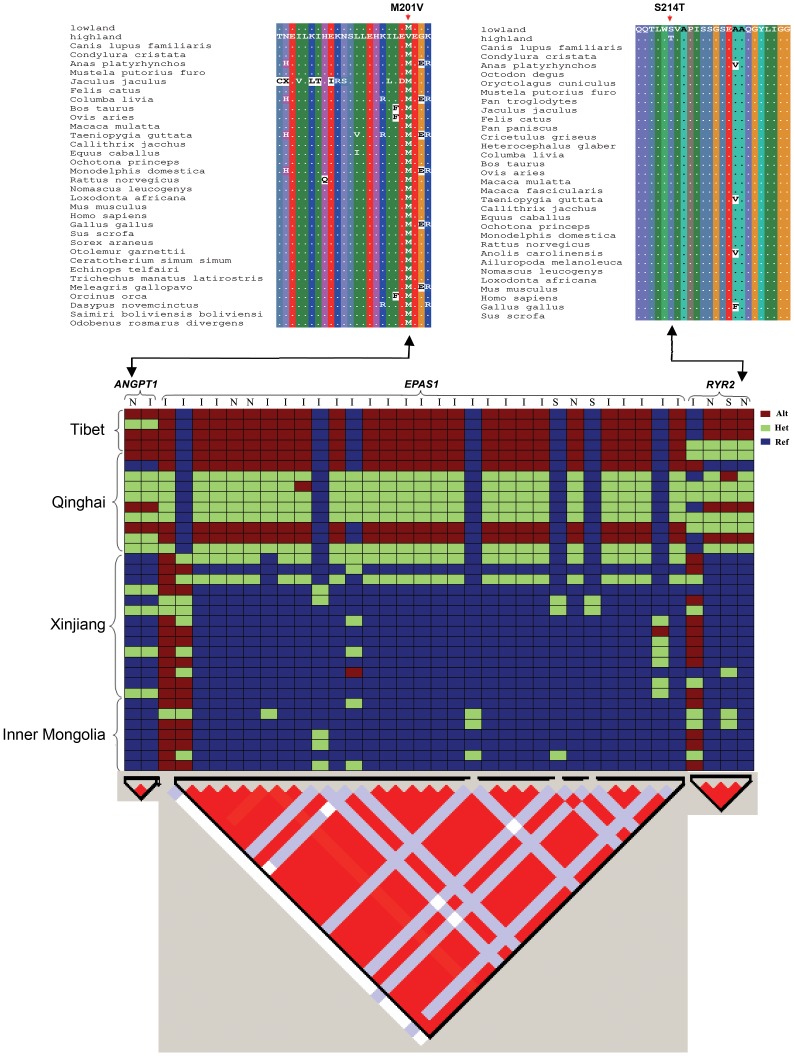
Top: Partial alignments of *ANGPT1* (left) and *RYR2* (right) amino acid sequences. The sequences with more than 15% gaps or less than 60% identity with lowland ortholog sequences were filtered out. Dots (.) represent residues identical to lowland wolves' sequence. The mutation S214T in *RYR2* and M201V in *ANGPT1* in highland wolves were denoted with arrows. Where highland showed the wolves from Tibet and Qinghai and lowland showed the wolves from Xinjiang and Inner Mongolia. Bottom: Genotypes plots of three hypoxic genes (top) and LD patterns (triangle plot, bottom). These genotypes derive from Sanger sequencing of 35 wolves and were encoded as homozygous reference (ref), heterozygote (het), and homozygous alternative (alt). Red regions represent a high degree of LD. I: intron; N: non-synonymous; S: synonymous.

A total of 6 nonsynonymous SNPs from 3 hypoxia-related genes (*EPAS1*, *RYR2*, and *ANGPT1*; ranging from 1 to 3 per gene) that were under selection at the top 5% level as indicated by F_ST_ and Δπ metrics were confirmed through re-sequencing (Fig. 5 and 7). All the 6 variants showed significantly different distributions of genotypes between highland and lowland wolves (Fig. 7 and Table S9, *P*≤3.57E-07). As defined by these nonsynonymous SNPs, two alleles from *RYR2* were only present in highland wolves (Fig. 7 and Table S9). In addition, one synonymous variant and one variant in an intron from *RYR2*, one variant in intron in *ANGPT1*, 21 of 26 variants in introns from *EPAS1*, also showed significantly different distributions of alleles and genotypes between highland and lowland wolves (Fig. 7 and Table S9, *P*≤8.01E-05).

Linkage disequilibrium (LD) analysis with the Haploview software [27] showed that the 3 hypoxia-related genes were located in single LD blocks (Fig. 7). Linkage analysis of re-sequenced genes showed complete linkage disequilibrium (r^2^ = 1) for 2 variants in *ANGPT1*, 23 variants in *EPAS1*, and 4 variants in *RYR2* (Fig. 7).

## Discussion

### Demography of the Tibetan grey wolf

Since the average altitude of Tibet plateau is substantially higher than our other localities, the more dramatic and extended population decline of Tibetan wolves may reflect more severe habitat loss there during the initial stages of glaciation [28]–[29]. Human migration into the Tibetan Plateau may have also contributed to the steeper decline of Tibetan wolves (Fig. 4). The earliest evidence of human occupation of the Tibetan Plateau consists of flakes and microliths found at 4500 to 5200 m in northern Tibet similar to those of northern Asian tool cultures dated 25,000 to 50,000 years ago [30]. Qi et al. [31] found that there have been two distinct, major prehistoric migrations of modern humans into the Tibetan Plateau. The first human migration occurred approximately 30,000 years ago followed by a migration 7–10 thousand years ago. The rapid growth of the human population on the Qinghai-Tibet Plateau could have resulted in the loss of habitats appropriate for large wildlife species as well as over exploitation, which then contributed to the population decline of the Tibetan grey wolves.

### Potential molecular mechanism of hypoxia adaptation

Positively selected hypoxia-related genes in highland wolves, identified with F_ST_ and Δπ metrics (Table S6 and S7), are enriched in the HIF signaling pathway (*P* = 1.57E-6), ATP binding (*P* = 5.62E-5), and the response to oxygen-containing compound (*P*≤5.30E-4). Specifically, 198 genes under selection (Table S6 and S7) were related to response to stimulus (Table S6 and S7) and the main stimulus in the high altitude population is low oxygen supply [1]. These categories appear to be biologically relevant to living at high altitudes by providing energy and oxygen for tissues and organs. In addition, given the population bottleneck experienced by the Tibetan wolf (Fig. 4), we expect average F_ST_ values may be inflated, however, this is not expected to produce locus specific effects on genes involved in the hypoxia pathway (e.g. [32]).

Combining results of F_ST_ and Δπ, 84 hypoxia-related genes appeared to be under selection (Table S7). To identify which of them could be responsible for local adaptation in the Tibetan plateau, we identified nonsynonymous SNPs (denoted “highland SNP”) in the genome data whose genotypes were homozygous reference in lowland wolves and homozygous alternative in Tibetan wolves (and either heterozygous or homozygous alternative in Qinghai wolves). Of the 84 genes, only three genes (*EPAS1*, *RYR2*, and *ANGPT1*) matched such criteria. Three highland SNPs were found in *EPAS1*, two in *RYR2*, and one in *ANGPT1* (Fig. 7). Moreover, SIFT, MAPP, and PolyPhen2 all identified that one of the two highland SNPs in *RYR2* (located in exon 8: chr04:2778722, Table S9) might affect protein function and resulted in a Ser-to-Thr (S214T) amino acid change in highland wolves. In addition, the highland SNP in *ANGPT1*, a T-to-C transition (SNP name: chr13_8141664; Table S9) found in exon 4, resulted in a Met-to-Val (M201V) amino acid change in highland wolves. The M201V mutation in *ANGPT1* was predicted to be benign by PolyPhen2, however, the same SNP was found to affect protein function with high probability in MAPP (MAPP score = 30.01, column score = 24.07, *P* = 2.924E-6) and with high confidence in SIFT. Multiple amino acid alignments of orthologs showed that these two amino acid variants (M201V and S214T) were only found in highland wolves (Fig. 7). Additional sequencing in a larger panel of wolves showed these highland SNPs in the above 3 genes had a frequency difference between highland and lowland wolves greater than 58% (*P*≤3.57E-07, Table S9; Fig. 7). However, analysis of these sequences for additional substitutions showed that some variants in these genes were in complete linkage disequilibrium (r^2^ = 1; Fig. 7) leaving open the possibly that high-altitude adaptation in the Tibetan wolves may involve multiple substitutions.

**Figure 8:**
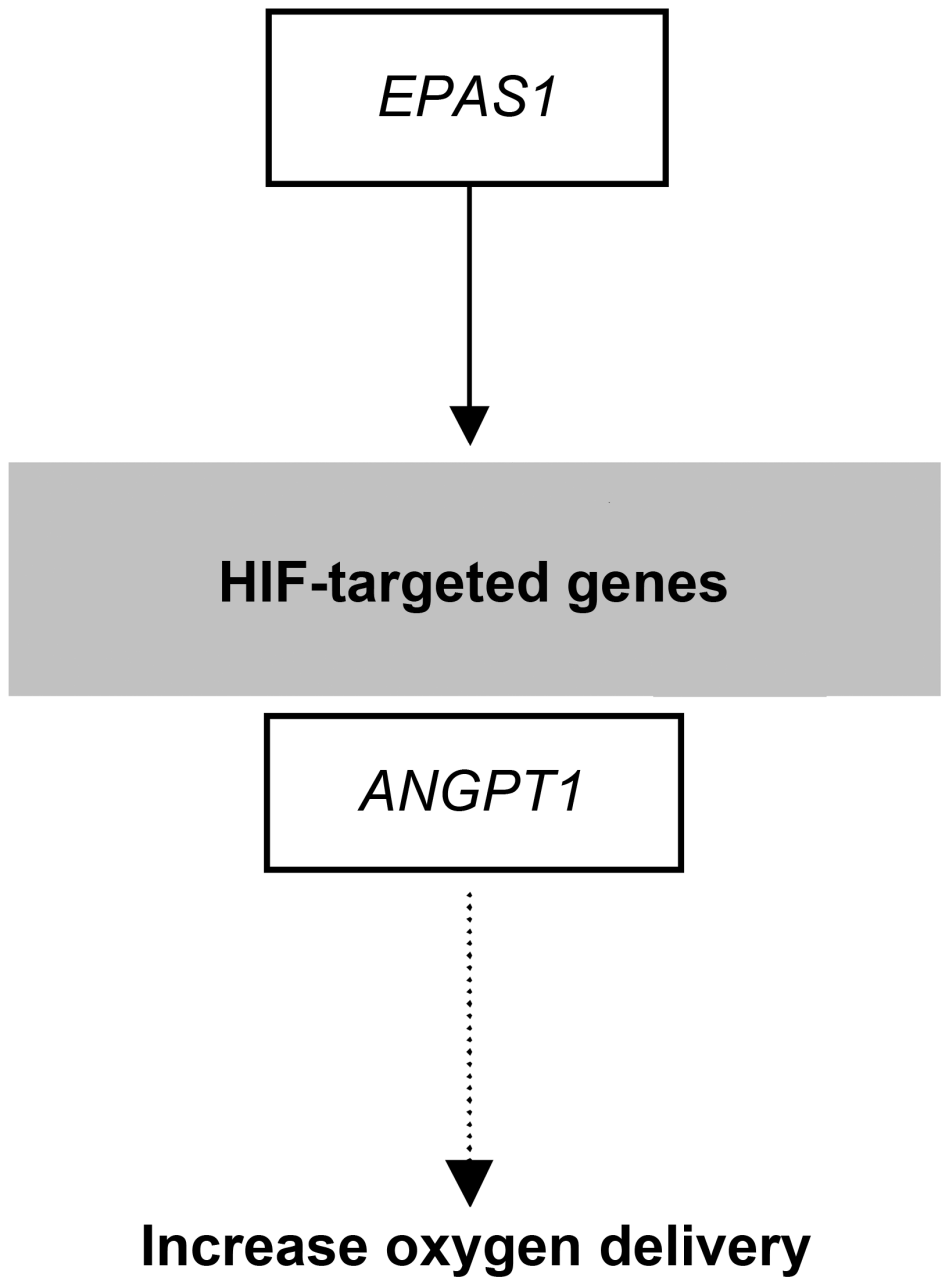
Selection candidates involved in the HIF pathway that were found to show evidence of positive selection in highland wolves. Solid lines indicate a direct relationship between enzymes and metabolites. Dashed lines indicate that more than one step is involved in the process. The genes outlined in black boxes were under selection and those indicated in gray boxes were provided for reference. See discussion of interactions in the text.

Gene function prediction showed that *EPAS1* and *ANGPT1* function in the HIF pathway were involved in the response to hypoxic stress (Fig. 8). *EPAS1* is a prime regulator during chronic hypoxia and directly regulates key genes such as erythropoietin (EPO), and vascular endothelial growth factor (VEGF) [33]–[34]. *ANGPT1* can increase tissue vascularization and result in increased oxygen delivery [35]. In addition, *RYR2* initiates cardiac excitation-contraction coupling by Ca^2+^-induced Ca^2+^ release [36] and some amino acid mutations in *RYR2* have been linked to heart failure in humans [37]. These three genes were found to be under selection for hypoxia adaptation in human populations living in high altitude. Specifically, *ANGPT1* was under positive selection in humans from Tibet [38], *EPAS1* was found to exhibit a significant signal for natural selection in humans from highland Qinghai-Tibet, the Andes, and Mongolia [3]–[10] and *RYR2* was under selection and within the top 0.1% of hits in high-altitude Ethiopian populations [39]. Consequently, highland wolves have evolved hypoxia adaptations at the molecular level in parallel with these human populations.

Finally, Ji et al. [9] found a significant increase in the frequencies of some hypoxia related alleles with increased-altitude in humans. In this study, we observed a similar trend in the *EPAS1* gene. Geographically, Qinghai appears as a transitional zone between Tibet and lowland (Xingjiang and Inner Mongolia) populations (Fig. 1). All individuals from Tibet are fixed for a single haplotype spanning the 31 variants of *EPAS1*, and four individuals from Qinghai are also fixed for this haplotype with the remaining individuals being heterozygous (Fig. 7). In contrast, in the low altitude populations of Xinjiang and Inner Mongolia, we observe lower heterozygosity and allele frequencies for this haplotype (Fig. 7; Table S9).

In conclusion, through the analysis F_ST_ and Δπ (Fig. 5) and re-sequencing (Fig. 7), we find consistent support for positive selection on three hypoxia-related genes in highland wolves of the Qinghai-Tibet Plateau. These genes potentially enhance function under hypoxic conditions by increasing oxygen delivery (*EPAS1* and *ANGPT1*; Fig. 8), or heart (*RYR2*) function. Given that these genes also appear under selection in high altitude populations of humans, a common genetic toolkit for rapid acclimation to hypoxia may be emerging. The grey wolf is a particularly dramatic example of the process of adaptation to high altitude environments given that high rates of migration among populations should stymie local adaptation. However, in grey wolves, dispersing individuals may select habitats similar to those they experienced during a prolonged maturation period in their natal pack [18]. Thus, despite the potential to disperse over large distances, fidelity to natal habitats may assist the process of local adaptation in wolves and other species.

## Materials and Methods

The experimental and analytical procedures used in this study are summarized in the flow chart in Figure S1.

### Samples

Blood samples were collected from 35 grey wolves from four distributions in China (Fig. 1 and Table S1). Of these, 30 individuals were verified as wild-born from a specific geographic locale and five individuals were captive-born from wild-born parents (Fig. 1 and Table S1). The latitude and longitude of wild-born parents was used for captive-born individuals. Samples were mapped with DIVA-GIS (version 7.5) (http://www.diva-gis.org/gdata) (Fig. 1). All activities followed the legal requirements and institutional guidelines set out by the government of P. R. China. Consent was given from all relevant institutions to obtain samples.

Genomic DNA from blood was isolated using a standard proteinase K digestion and phenol-chloroform extraction procedure [40].

### Genome sequencing

Twenty-six polymorphic microsatellite loci (Table S10) were used to analyze the genetic structure of the 35 Chinese wolves with previously published procedures [41]. We performed a series of independent runs using population clusters (K) from 1 to 10, assuming an admixture model and with burn-in and replication values set at 50,000 and 1,000,000, respectively, in STRUCTURE [42]. We ran three independent simulations for each K value and checked the consistency of results.

In addition, the 35 Chinese wolves were genotyped on a MassARRAY for single nucleotide polymorphism. MALDI-TOF mass spectrum genotyping were evaluated to determine if some of them had significant domestic dog admixture/ancestry by analysis of 25 of the top 27 dog-wolf ancestry informative markers (AIM) identified in vonHoldt et al [43] through pairwise F_ST_ comparison of 912 domestic dogs and 155 grey wolves. Three of the 25 AIM markers were polymorphic in the panel of wolves and not useful for admixture/ancestry analysis (Table S11). Based on the final panel of 22 AIM markers, 4 of the wolves had <90% of the respective diagnostic wolf alleles and were thus considered to have significant dog admixture/ancestry and eliminated from the genomic analyses (Table S11).

Based on the above microsatellite and AIM data (Fig. S2; Table S10 and S11), eight individuals (Fig. 1 and S2; Table S1) that represented four distinct populations from lowland (Xinjiang and Inner Mongolia) and highland (Tibet and Qinghai) regions were chosen for complete genome sequencing at BGI, Shenzhen, China. In addition, short reads of an additional sample (RKWL) from Inner Mongolia in a previous genome study [21] were processed together with the eight wolves.

### Mapping and genotyping

All short reads were aligned to the dog genome (boxer genome, CanFam3.1) using Bowtie2 [44] under the local alignment algorithm with very sensitive model settings and proper insert sizes. Other parameters were set as default.

After mapping the short reads to the reference genome, we applied two major tools, Picard (http://picard.sourceforge.net) and Genome Analysis Toolkit (GATK) toolset [45], to process the alignments in order to perform genotype calls. The whole pipeline converted the short reads to bam format alignment files [46], and genotype calls were placed in a vcf format (http://www.1000genomes.org/node/101) from bam files after multiple steps (Fig. S3). We describe the details of our pipeline in a supplementary file (Protocol S1). After producing the genotype calls from GATK, we applied several conservative data quality filters to further control the data quality, grouped into two levels: genome filters (GF, which was based on the reference genome's features and polymorphism across samples) and sample filters (SF, which was based on the genotype calls of each sample). We describe the details of the filters in a supplementary file (Protocol S2).

### Validation of SNP calls

The Ti/Tv for all the nine Chinese wolves was close to ∼2.3 with a mean of 2.34 (range: 2.321–2.355; Table S12), which was similar to other complete genome sequencing studies [45], [47]–[48].

Furthermore, we compared our genome data with the Illumina SNP chip calls of RKWL [21], which included more than 170,000 high quality markers throughout the genome. All three types of genotype calls had very high concordance (homozygous reference: 99.77%; heterozygous: 99.82%; homozygous non-reference: 99.97%). In addition, a total of 17,210 bp DNA sequences from 17 genes (Table S9) were used to check the genotype calls of the remaining eight genomes in this study (Table S1). Ninety SNPs from the 17,210 bp DNA sequences were found in the eight complete genomes. Among all 720 genotypes from the 90 SNPs, only 2 genotypes in one individual (IM07) were not found in re-sequencing data.

### Gene annotation and hypoxia genes list of dog

We used publicly available information regarding gene locations in the dog genome (canFam3.1) to build a comprehensive set of transcribed and coding regions. Gene coordinates were retrieved from Ensembl (release 70, downloaded on Feb 11th 2013 from ftp://ftp.ensembl.org/pub/release-70/gtf/canis_familiaris/Canis_familiaris.CanFam3.1.70.gtf.gz) and NCBI (ftp://ftp.ncbi.nih.gov/genomes/Canis_lupus_familiaris/GFF/ref_CanFam3.1_top_level.gff3.gz, downloaded on Feb 11th 2013). The total set comprised 28,538 genes and 51,781 transcripts, many of them redundant between the two annotation sources. We considered as duplicated entries genes that: 1) had overlapping coordinates and; 2) had similar gene names or symbols; or 3) had any of their transcripts sharing more than 60% of the exons (corresponding to the threshold used by NCBI in a similar NCBI/Ensembl matching). Transcripts with the exact same exon coordinates were considered duplicates, while transcripts with partial differences were considered alternative transcripts of the same gene.

We also tested the transcripts for apparently intact open reading frames: proper start and stop codons, a coding region multiple of 3 bp and no in-frame stop codons. Finally, the complete set of non-redundant coding regions with apparent intact coding frames (‘unique CDS OK’ set) used for our analyses had 30,533 transcripts corresponding to 21,108 genes.

In order to build a comprehensive set of annotated hypoxia-related genes in the domestic dog, we searched the available information from four different sources with keywords “hypoxia” and “HIF” (Hypoxia-Inducible Factors pathway) in CanFam3.1 on the UCSC genome browser database [49] (http://genome.ucsc.edu/cgi-bin/hgGateway?hgsid=323162145), Ensembl [50] (http://www.ensembl.org/Canis_familiaris/Info/Index), SeqGene files from the NCBI database (http://www.ncbi.nlm.nih.gov/gene), and UniProt [51] (http://www.uniprot.org/). We also downloaded genes associated with Gene Ontology annotation terms [52] (http://www.geneontology.org/) and KEGG pathways [53] (http://www.kegg.jp) via Entrez Gene that may be involved in a hypoxia response via HIF activation with keywords “hypoxia” and “HIF”. These methods identified 534 genes.

Moreover, in order to obtain a maximal extensive annotation of the genomic complement of hypoxia-related genes in dog, we searched for hypoxia-related genes in humans from RefSeq [54], KnownGene [55] and VEGA [56], and then mapped them to dog genome using BLASTP for homology-based gene prediction. The alignments shorter than 150 bp or the target sequences with no chromosome locations in CanFam3.1 were discarded. Moreover, some putative hypoxia-related genes in human [5], [9] were also used to search for their homologs in the dog genome. Since it is difficult to find univocal chromosome locations in the dog genome for the homologous microRNAs between human and dog, the candidate list of hypoxia genes did not include microRNAs. Similarly, potential candidate genes identified in the mitochondrial genome and on the X chromosome were not considered in this study.

### Population structure from genome data

The SNPs of the nine Chinese wolves were pruned to remove SNPs with high pairwise genotypic association (r^2^) for a proper use in principal components analysis (PCA using Eigensoft) and Bayesian clustering analysis (using STRUCTURE).

Highly linked SNPs with r^2^>0.2 were removed from the dataset of variant calls using PLINK [57] with the setting “indep-pairwise 50 5 0.2”. Then, the pruned SNPs dataset was used for the Bayesian inference program STRUCTURE (v2.3.4 [42]) to assess genetic admixture of the nine Chinese wolves.

We utilized 10,000 burn-in iterations and 10,000 MCMC iterations in STRUCTURE (v2.3.4), with three repetitions of these parameter settings for each number of K populations interrogated. The alpha and likelihood statistics were monitored and verified to reach convergence before both the 10,000 burn-in and 10,000 MCMC iterations were completed during each repetition for each number of K populations analyzed. We compared likelihood values (averaged over 3 runs) for each K value assessed, and the parameter Δ [58] for K = 1 to 5 with the cluster assignment results. Moreover, to visualize the dominant relationships in the merged SNP dataset of nine Chinese wolves, we used the *smartpca* program distributed in the Eigensoft package for principal component analysis (PCA [59]).

### Demography from genome data

We used the pairwise sequentially markovian coalescent (PSMC [22]) to infer the demographic history of all the nine Chinese wolves. Briefly, the method uses the distribution of heterozygote sites across the genome and a Hidden Markov Model to reconstruct the history of effective population sizes. The following parameters were used: numbers of iterations = 25, time interval = 64*1, mutation rate per generation = 1.0×10^−8^ and generation time = 3.

### Genome-wide selection scans

Evidence for selection was evaluated by contrasting F_ST_ and Δπ calculated from for the genome sequences of highland and lowland wolves [60]–[62]. The highland wolves included two individuals from Tibet and two from Qinghai and the lowland wolves included five individuals from Inner Mongolia and Xinjiang, groupings consistent with population structure analyses (Fig. 1 and 3). For calculations of F_ST_ and Δπ, we used a sliding window approach in which we divided the reference genome into overlapping windows of size 100 kb with 20 kb increments. For each 100 kb-window, we computed summary statistics using only sites that pass the GF2 filter (Protocol S2) and where genotypes were observed and pass SF in all wolves (Protocol S2).

For each summary statistic, we computed empirical percentiles by ranking each window for F_ST_ and Δπ and transforming the ranks to percentiles (% F_ST_ and % Δπ). We then calculated a “joint” empirical percentile (% Joint) (1) by computing the product of the empirical percentiles obtained for the two summary statistics in each window [(% Product) = (% F_ST_)*(% Δπ)] and (2) ranking each window by the products (% Product) and transforming the ranks to percentiles (% Joint).

For each metric, we defined the windows with F_ST_>0.259 and Δπ>5.311 (corresponding to top 5% level for joint empirical percentile) as *outlier windows*. Since the outlier windows are often clustered together in the genome, we joined outlier windows and intervening sequence to define *outlier regions* when windows were found within 200 kb of each other.

### Gene enrichment

The set of genes from our selection hits were tested for significant enrichment in Gene Ontology (GO) categories, Kegg/Reactome pathways (KGR) and Human Phenotype Ontologies (HPO) using the online tool g:Profiler [63] (http://biit.cs.ut.ee/gprofiler/). All genes of dog annotated in Ensembl were used as background set, and the Benjamini-Hochberg false discovery rate [64] was applied to correct for multiple testing. We only reported significantly enriched categories that included ≥5 genes and with multiple testing corrected *P* value≤0.05.

### Prediction of functional variation

For functional prediction of non-synonymous coding SNPs, we focused on protein sequences whose mutations had significant differences at the 5% level in the distributions of genotypes between highland and lowland wolves, tested by the association test in the Haploview software [27]. Specifically, individuals from Tibet had to be all homozygous alternatives, whereas at least three homozygous reference and no homozygous alternatives had to be found in lowland wolves. However, the population structure analysis based on genome wide SNPs showed that the two Qinghai wolves were closer to Tibet wolves or intermediate (Fig. 3), which is consistent with their geography (Fig. 1). Consequently, no homozygous reference genotypes found in Qinghai wolves were used to identify SNPs with the significant difference at the 5% level between the highland and lowland wolves.

We identified orthologs through protein BLAST search in GenBank and multi-protein sequence alignment in MUSCLE [65] for prediction of functional variation with Multivariate Analysis of Protein Polymorphism (MAPP [25]). An alignment with more than 10% gaps or less than 60% identity between each protein and its lowland ortholog was considered a different form of transcript or false annotation [66].

SIFT [24], MAPP [25], and PolyPhen2 [26] were used to predict the putatively functional importance of non-synonymous coding SNPs. Thresholds in determining whether a given metric predicted these SNPs to be functional were as follows: PolyPhen2 “PROBABLY/POSSIBLE DAMAGING”, SIFT “AFFECT PROTEIN FUNCTION”, and MAPP “Bad Amino Acids”.

### Causal mutation validation

In order to further identify potential targets of selection, a set of SNPs and associated genomic sequences were re-sequenced in additional wolf samples. Three criteria were used to select these SNPs: (1) the genes should be listed in hypoxia-related gene candidate list and identified as outliers by Δπ and F-statistic; (2) the distributions of selected alleles had to be highly differentiated between lowland and highland groups as in the paragraph “Prediction of functional variation”, but homozygous references in all lowland wolves; and (3) the selected alleles must have biological effects. Based on these three criteria, 6 non-synonymous SNPs from 3 hypoxia-related genes (*ANGPT1*, *EPAS1*, and *RYR2*; Table S8; Fig. 5 and 7) were used for validation in an extended study involving all 35 individuals. Moreover, 14 non-synonymous SNPs from 11 hypoxia-related genes and 10 SNPs located at 5′ UTR (untranslated region) or 3′ UTR from 3 hypoxia-related genes were used for validating the 8 genome data (excluding RKWL) by Sanger sequencing (Table S8).

Primer sets (Table S8) for amplifying the target sequences were designed based on the dog *de novo* assembly (CanFam3.1). After PCR, all products were subsequently sequenced using an ABI 3730XL (Applied Biosystems).

Linkage disequilibrium (LD), chi-square and p-values for the allele frequencies in highland (Tibet + Qinghai) vs. lowland (Xinjiang + Inner Mongolia) wolves for the re-sequenced SNPs from the 3 hypoxia-related genes (*ANGPT1*, *EPAS1*, and *RYR2*) were assessed with the Haploview program [27].

## Supporting Information

Figure S1 The flow chart of the experimental and analytical procedures used in this study.
(TIF)

Figure S2 Assignment of individual microsatellite genotypes to populations (K = 2–6) in STRUCTURE with 35 Chinese wolves. The highest probability assignment is *K = *6. Colors represent the proportion of individual genotypes assign to one of *K* clusters.
(TIF)

Figure S3 The overview of genotyping pipeline in this study.
(TIF)

Table S1 Samples in this study.
(DOC)

Table S2 Descriptive statics for the complete genome data used in this study.
(DOC)

Table S3 Summary of useable sites that pass the GF2 and SF filters in the nine Chinese wolves. The proportion of the covered genome is based on the non-N reference size (2,194,412,237).
(DOC)

Table S4 Summary of useable sites that pass the GF1 and SF filters in the nine Chinese wolves. The proportion of covered genome is based on the non-N reference size (2,194,412,237).
(DOC)

Table S5 *A priori* hypoxia related gene list.
(XLS)

Table S6 GO analysis of the 1548 genes identified with F_ST_ and Δπ.
(XLS)

Table S7 GO analysis of the 84 hypoxia-related genes identified with F_ST_ and Δπ.
(XLS)

Table S8 The SNPs for PCR amplification and sequencing.
(DOC)

Table S9 The association test with HaploView. The SNP names were combined with chromosome and physical position.
(DOC)

Table S10 Microsatellite loci in this study.
(DOC)

Table S11 The genotype and analysis of 25 of top 27 dog-wolf ancestry informative markers in 35 Chinese wolves.
(XLS)

Table S12 Transitions: transversions ratio (Ti:Tv) for each individual using sites pass GF2 and SF filters.
(DOC)

Protocol S1 Genotyping pipeline.
(DOCX)

Protocol S2 Post genotype filters.
(DOCX)
