# Supplementary figures and images for: Hypoxia Adaptations in the Grey Wolf (Canis lupus chanco) from Qinghai-Tibet Plateau
Source: PLoS Genet. 2014 Jul 31;10(7):e1004466. doi: 10.1371/journal.pgen.1004466 (PMC4117439; doi:10.1371/journal.pgen.1004466)

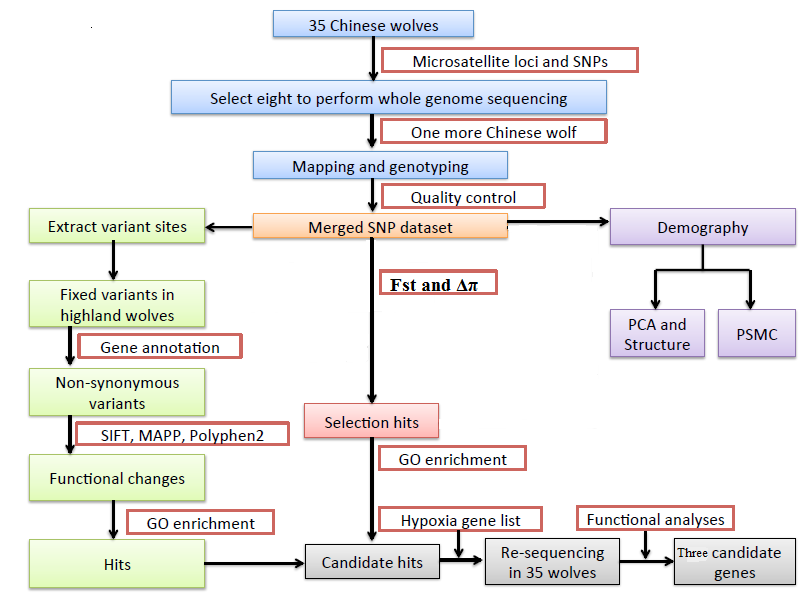

Supplement: Figure S1 — The flow chart of the experimental and analytical procedures used in this study. (TIF) [file pgen.1004466.s001.tif]

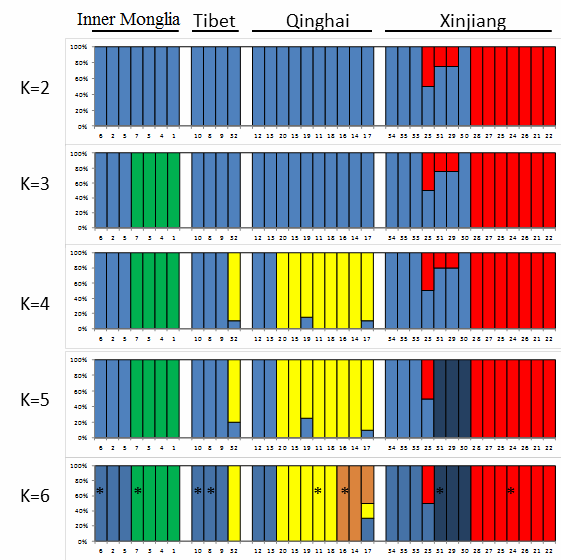

Supplement: Figure S2 — Assignment of individual microsatellite genotypes to populations (K = 2–6) in STRUCTURE with 35 Chinese wolves. The highest probability assignment is K = 6. Colors represent the proportion of individual genotypes assign to one of K clusters. (TIF) [file pgen.1004466.s002.tif]

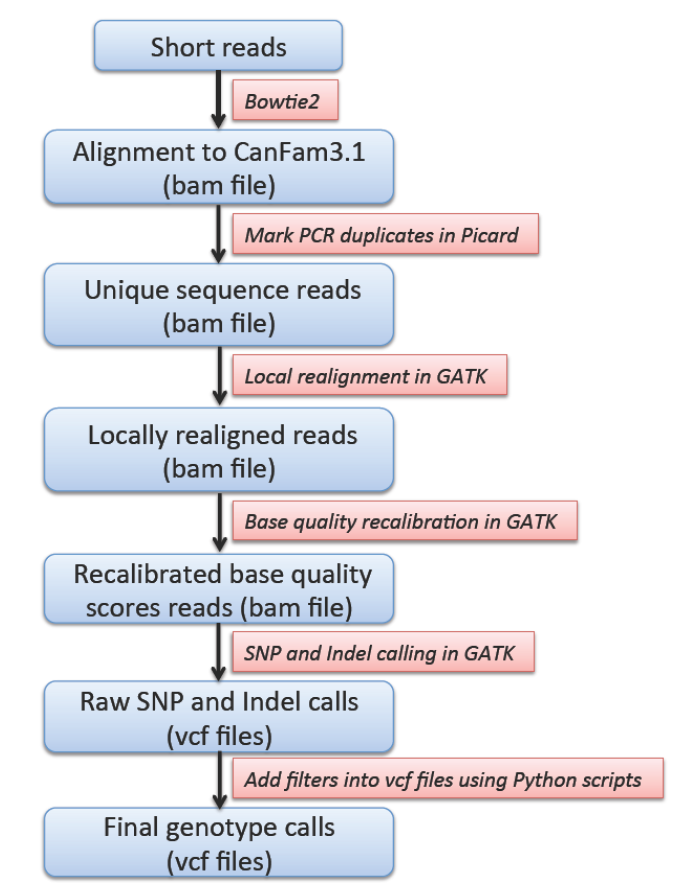

Supplement: Figure S3 — The overview of genotyping pipeline in this study. (TIF) [file pgen.1004466.s003.tif]
