# Supplementary material for: Hypoxia Adaptations in the Grey Wolf (Canis lupus chanco) from Qinghai-Tibet Plateau
Source: PLoS Genet. 2014 Jul 31;10(7):e1004466. doi: 10.1371/journal.pgen.1004466 (PMC4117439; doi:10.1371/journal.pgen.1004466)
Supplement: Table S1 — Samples in this study. (DOC) [file pgen.1004466.s004.doc]

Table S1: Samples in this study.

| Group | Code | Locale of Origin in China | Source | Birth Status |
| --- | --- | --- | --- | --- |
| Lowland | IM01 | Inner Mongolia | Haerbing Zoo | wild born |
|  | IM02 | Inner Mongolia | Haerbing Zoo | captive born |
|  | IM03 | Inner Mongolia | Haerbing Zoo | wild born |
|  | IM04 | Inner Mongolia | Haerbing Zoo | captive born |
|  | IM05 | Inner Mongolia | Haerbing Zoo | captive born |
|  | IM06* | Inner Mongolia | Haerbing Zoo | wild born |
|  | IM07* | Inner Mongolia | Haerbing Zoo | wild born |
|  | XJ21 | Xinjiang | Kalamaili Nature Reserve | wild born |
|  | XJ22 | Xinjiang | Kalamaili Nature Reserve | wild born |
|  | XJ23 | Xinjiang | Kalamaili Nature Reserve | wild born |
|  | XJ24* | Xinjiang | Kalamaili Nature Reserve | wild born |
|  | XJ25 | Xinjiang | Kalamaili Nature Reserve | wild born |
|  | XJ26 | Xinjiang | Kalamaili Nature Reserve | captive born |
|  | XJ27 | Xinjiang | Kalamaili Nature Reserve | wild born |
|  | XJ28 | Xinjiang | Kalamaili Nature Reserve | wild born |
|  | XJ29 | Xinjiang | Duzhishan Garden | wild born |
|  | XJ30* | Xinjiang | Duzhishan Garden | wild born |
|  | XJ31 | Xinjiang | Kuitun Garden | wild born |
|  | XJ33 | Xinjiang | Kashi Garden | wild born |
|  | XJ34 | Xinjiang | Kashi Garden | wild born |
|  | XJ35 | Xinjiang | Kashi Garden | wild born |
| Highland | TI08 | Tibet | Luobulingka Zoo | wild born |
|  | TI09* | Tibet | Luobulingka Zoo | wild born |
|  | TI10 | Tibet | Luobulingka Zoo | wild born |
|  | TI32* | Tibet | Kashi Garden | wild born |
|  | QH11* | Qinghai | Xining Zoo | wild born |
|  | QH12 | Qinghai | Xining Zoo | wild born |
|  | QH13 | Qinghai | Xining Zoo | wild born |
|  | QH14 | Qinghai | Xining Zoo | wild born |
|  | QH15 | Qinghai | Xining Zoo | captive born |
|  | QH16* | Qinghai | Xining Zoo | wild born |
|  | QH17 | Qinghai | Xining Zoo | wild born |
|  | QH18 | Qinghai | Xining Zoo | wild born |
|  | QH19 | Qinghai | Xining Zoo | wild born |
|  | QH20 | Qinghai | Xining Zoo | wild born |

* denoted these samples for genome sequencing.
