# Supplementary material for: Hypoxia Adaptations in the Grey Wolf (Canis lupus chanco) from Qinghai-Tibet Plateau
Source: PLoS Genet. 2014 Jul 31;10(7):e1004466. doi: 10.1371/journal.pgen.1004466 (PMC4117439; doi:10.1371/journal.pgen.1004466)
Supplement: Table S2 — Descriptive statics for the complete genome data used in this study. (DOC) [file pgen.1004466.s005.doc]

Table S2: Descriptive statics for the complete genome data used in this study.

| **Samples** | **Total**  **reads** | **Covered Bases (Gb)** | **Overall alignment** | | **% PCR duplicates** | **Mean coverage** |
| --- | --- | --- | --- | --- | --- | --- |
|  |  |  | **Reads** | **Rate** |  |  |
| IM06 | 761280586 | 68.52 | 752226946 | 98.81% | 0.114885 | 25.67 X |
| IM07 | 719010666 | 64.71 | 708801794 | 98.58% | 0.159551 | 22.93 X |
| XJ24 | 686608622 | 61.79 | 676862258 | 98.58% | 0.069318 | 24.29 X |
| XJ30 | 757945292 | 68.21 | 747680654 | 98.65% | 0.068192 | 26.87 X |
| QH11 | 763248046 | 68.69 | 752432134 | 98.58% | 0.106189 | 25.93 X |
| QH16 | 759371772 | 68.34 | 750377280 | 98.82% | 0.086161 | 26.44 X |
| TI09 | 756115520 | 68.05 | 746814582 | 98.77% | 0.100641 | 25.89 X |
| TI32 | 750411736 | 67.53 | 740532644 | 98.68% | 0.094088 | 25.85 X |
| RKWL | 694048646 | 61.3^#^ | 685775374 | 98.81% | 0.066873 | 27.43 X |

# This is the value for uniquely aligned bases.
