# Supplementary material for: Hypoxia Adaptations in the Grey Wolf (Canis lupus chanco) from Qinghai-Tibet Plateau
Source: PLoS Genet. 2014 Jul 31;10(7):e1004466. doi: 10.1371/journal.pgen.1004466 (PMC4117439; doi:10.1371/journal.pgen.1004466)
Supplement: Table S3 — Summary of useable sites that pass the GF2 and SF filters in the nine Chinese wolves. The proportion of the covered genome is based on the non-N reference size (2,194,412,237). (DOC) [file pgen.1004466.s006.doc]

Table S3: Summary of useable sites that pass the GF2 and SF filters in the nine Chinese wolves. The proportion of covered genome is based on the non-N reference size (2,194,412,237).

| **Sample** | **Homozygous reference** | **SNPs** | | **Total useable sites** | **Proportion of covered genome** | **Hetero-**  **zygosity** | **SNP rate** |
| --- | --- | --- | --- | --- | --- | --- | --- |
|  |  | **Total** | **Hetero-**  **zygous** |  |  |  |  |
| RKWL | 1382096535 | 3220666 | 1680523 | 1385317201 | 63.13% | 0.001213 | 0.002325 |
| IM06 | 1371571136 | 3272754 | 1878046 | 1374843890 | 62.65% | 0.001366 | 0.002381 |
| IM07 | 1356542901 | 3317662 | 2047522 | 1359860563 | 61.97% | 0.001506 | 0.002439 |
| QH11 | 1376271717 | 3332101 | 1868265 | 1379603818 | 62.87% | 0.001354 | 0.002415 |
| QH16 | 1369566458 | 3229216 | 1673191 | 1372795674 | 62.56% | 0.001219 | 0.002352 |
| TI09 | 1367586421 | 2925783 | 965935 | 1370512204 | 62.45% | 0.000705 | 0.002135 |
| TI32 | 1369680772 | 3010114 | 1183656 | 1372690886 | 62.55% | 0.000862 | 0.002193 |
| XJ24 | 1367921643 | 3440638 | 2190052 | 1371362281 | 62.49% | 0.001597 | 0.002509 |
| XJ30 | 1371497846 | 3482449 | 2243323 | 1374980295 | 62.66% | 0.001632 | 0.002533 |
