# Supplementary material for: Hypoxia Adaptations in the Grey Wolf (Canis lupus chanco) from Qinghai-Tibet Plateau
Source: PLoS Genet. 2014 Jul 31;10(7):e1004466. doi: 10.1371/journal.pgen.1004466 (PMC4117439; doi:10.1371/journal.pgen.1004466)
Supplement: Table S4 — Summary of useable sites that pass the GF1 and SF filters in the nine Chinese wolves. The proportion of covered genome is based on the non-N reference size (2,194,412,237). (DOC) [file pgen.1004466.s007.doc]

Table S4. Summary of useable sites that pass the GF1 and SF filters in the nine Chinese wolves. The proportion of covered genome is based on the non-N reference size (2,194,412,237).

| **Samples** | **Non-variant**  **sites** | **SNPs** | **Total**  **useable sites** | **Proportion of**  **covered genome** |
| --- | --- | --- | --- | --- |
| RKWL | 1354867338 | 2312319 | 1357179657 | 61.85% |
| IM06 | 1346297548 | 2368404 | 1348665952 | 61.46% |
| IM07 | 1332266092 | 2415566 | 1334681658 | 60.82% |
| QH11 | 1350610016 | 2403582 | 1353013598 | 61.66% |
| QH16 | 1344398778 | 2334708 | 1346733486 | 61.37% |
| TI09 | 1341763025 | 2108604 | 1343871629 | 61.24% |
| TI32 | 1344447029 | 2176988 | 1346624017 | 61.37% |
| XJ24 | 1341993905 | 2479613 | 1344473518 | 61.27% |
| XJ30 | 1345352954 | 2506914 | 1347859868 | 61.42% |
