# Supplementary material for: Hypoxia Adaptations in the Grey Wolf (Canis lupus chanco) from Qinghai-Tibet Plateau
Source: PLoS Genet. 2014 Jul 31;10(7):e1004466. doi: 10.1371/journal.pgen.1004466 (PMC4117439; doi:10.1371/journal.pgen.1004466)
Supplement: Table S8 — The SNPs for PCR amplification and sequencing. (DOC) [file pgen.1004466.s011.doc]

Table S8 The SNPs for PCR Amplification and Sequencing.

| Gene &  amplicon | Regions  covered  size (bp) | No. SNPs covered^1^ | | | | | | | Forward primer (5’ to 3’) | Reverse primers (5’ to 3’) |
| --- | --- | --- | --- | --- | --- | --- | --- | --- | --- | --- |
|  |  | In- | | Syn- | | Non- | | UTRs |  |  |
| ANGPT1 | 833 | 1 | 0 | | 1 | | 0 | | CTTGTATATGAGATGTACCCCA | CTACATTTAAGTGTCTAACAGCAT |
| EPAS1 |  | | | | | | | | | |
| EPAS1-1 | 966 | 18 | 2 | | 1 | | 0 | | CTTTGCCGTTACTGTCTGC | TCTCATCCCTGTTCTTATCATTT |
| EPAS1-2 | 898 | 8 | 0 | | 2 | | 0 | | CTTTGCCGTTACTGTCTGC | TCTCATCCCTGTTCTTATCATTT |
| RYR2 |  | | | | | | | | | |
| RYR2-1 | 854 | 1 | 1 | | 1 | | 0 | | GTGCTGGCTAYTACGACTT | CAACTATTATCCATTGACTCCT |
| RYR2-2 | 792 | 0 | 0 | | 1 | | 0 | | TGCAGATAAAAGTTCCCAAAG | GTAGCATGTAAAGCACGCAC |
| ROS1 | 685 | 1 | 1 | | 2 | | 0 | | AGCCGACAGTAGCCTCAAG | GCAGTTATGGGGATGGTCA |
| CYP2B6 |  | | | | | | | | | |
| CYP2B6-1 | 837 | 5 | 2 | | 1 | | 0 | | AGGAAATATGGGTCTGTAATCAC | TGGTAAATCCTCCAGAGTTAG |
| CYP2B6-3 | 853 | 3 | 0 | | 1 | | 0 | | GGTGGTTCAGGGTAGGTCC | CCCGCATAGCCATCACA |
| BACH1 | 729 | 3 | 0 | | 1 | | 0 | | CAAGGGTCAGGGGAACTA | AAATTAAAATCAGGGTGACTT |
| NR3C1 | 881 | 0 | 2 | | 1 | | 0 | | CTGTCCAAGGCAGTTTCAC | TGGCACCTATTCCAATTTTC |
| COL11A1 | 801 | 1 | 0 | | 1 | | 0 | | CTTTCAGGTTGAGGCAAG | ATGAAACAACTTATAATACCTTTG |
| COL24A1 | 768 | 0 | 2 | | 2 | | 0 | | ATGAAACAGCGTTGTCGC | TACGGGAGGTCCAGGGT |
| ITPR2 | 836 | 2 | 1 | | 1 | | 0 | | AGGTTTAGACTTTCTTCCACTTATT | CTCCTTTCCCCATCTGACTC |
| PXN | 809 | 0 | 1 | | 1 | | 0 | | GTGACAGCCCGCAGAAG | GAGGGAATCGGTGACAACT |
| WTIP | 787 | 2 | 1 | | 2 | | 0 | | ACCCTGAAAGAGTTTAGAATAGAT | TCTCATCCAAGAGGAGCAA |
| HIF3A | 950 | 12 | 1 | | 0 | | 0 | | AAGATGACTCGCTGGTTTG | GCTCTGGTGGACTGGATAA |
| NOX3 | 1004 | 7 | 0 | | 1 | | 0 | | TTATTCTGGGTGTAAGTAGAGTTG | TAGTATAGTTCCCTGACCATAAGT |

1. 1. In- = Intron; Syn- = synonymous; Non- = nonsynonymous

Continued Table 11:

| Gene &  amplicon | Regions  covered  size (bp) | No. SNPs covered^1^ | | | | Forward primer (5’ to 3’) | Reverse primers (5’ to 3’) |
| --- | --- | --- | --- | --- | --- | --- | --- |
|  |  | In- | Syn- | Non- | UTRs |  |  |
| SLC25A37 | 1007 | 0 | 0 | 0 | 3* | TTCTCACCAAGCACCAGC | ACTCGCATCATTCACAACG |
| CD36 | 970 | 0 | 0 | 0 | 3** | CCYCCGATACAACCAGTGC | ATCTTCTTGTGATTTACGCTTT |
| CALCRL | 950 | 0 | 0 | 0 | 4** | AATATCATAGCCAGTGACTATTTG | GGTCTATGTCCTCTGCCCT |

1. 1. In- = Intron; Syn- = synonymous; Non- = nonsynonymous; *: 3’ UTR; **: 5’ UTR
