# Supplementary material for: Hypoxia Adaptations in the Grey Wolf (Canis lupus chanco) from Qinghai-Tibet Plateau
Source: PLoS Genet. 2014 Jul 31;10(7):e1004466. doi: 10.1371/journal.pgen.1004466 (PMC4117439; doi:10.1371/journal.pgen.1004466)
Supplement: Table S9 — The association test with HaploView. The SNP names were combined with chromosome and physical position. (DOC) [file pgen.1004466.s012.doc]

Table S9: The association test with HaploView. The SNP names were combined with chromosome and physical position.

| Gene | Type | SNP names | Allele type | Highland, lowland  frequencies | Chi-square | *P* value |
| --- | --- | --- | --- | --- | --- | --- |
| ANGPT1 | Nonsynonymous | chr13_8141664 | Alt | 0.679, 0.095 | 25.913 | 3.57E-07 |
|  | Intron | chr13_8141820 | Alt | 0.679, 0.095 | 25.913 | 3.57E-07 |
| EPAS1 | Intron | chr10:48629843 | Alt | 0.214, 0.071 | 3.06 | 0.0802 |
|  | Intron | chr10:48629865 | Alt | 1.000, 0.214 | 41.622 | 1.11E-10 |
|  | Intron | chr10:48629952 | Alt | 0.786, 0.048 | 40.622 | 1.85E-10 |
|  | Intron | chr10:48630008 | Alt | 0.786, 0.048 | 40.622 | 1.85E-10 |
|  | Nonsynonymous | chr10:48630137 | Alt | 0.786, 0.048 | 40.622 | 1.85E-10 |
|  | Nonsynonymous | chr10:48630153 | Alt | 0.786, 0.048 | 40.622 | 1.85E-10 |
|  | Intron | chr10:48630311 | Alt | 0.786, 0.024 | 44.206 | 2.96E-11 |
|  | Intron | chr10:48630313 | Alt | 0.786, 0.048 | 40.622 | 1.85E-10 |
|  | Intron | chr10:48630420 | Alt | 0.821, 0.048 | 43.815 | 3.61E-11 |
|  | Intron | chr10:48630422 | Alt | 1.000, 0.881 | 3.59 | 0.0581 |
|  | Intron | chr10:48632729 | Alt | 0.786, 0.048 | 40.622 | 1.85E-10 |
|  | Intron | chr10:48632825 | Alt | 0.214, 0.190 | 0.06 | 0.8073 |
|  | Intron | chr10:48632834 | Alt | 0.786, 0.048 | 40.622 | 1.85E-10 |
|  | Intron | chr10:48632852 | Alt | 0.786, 0.048 | 40.622 | 1.85E-10 |
|  | Intron | chr10:48632880 | Alt | 0.786, 0.048 | 40.622 | 1.85E-10 |
|  | Intron | chr10:48632915 | Alt | 0.786, 0.048 | 40.622 | 1.85E-10 |
|  | Intron | chr10:48633040 | Alt | 0.786, 0.048 | 40.622 | 1.85E-10 |
|  | Intron | chr10:48633107 | Alt | 0.786, 0.048 | 40.622 | 1.85E-10 |
|  | Intron | chr10:48633165 | Alt | 1.000, 0.929 | 2.09 | 0.1483 |
|  | Intron | chr10:48633207 | Alt | 0.786, 0.048 | 40.622 | 1.85E-10 |
|  | Intron | chr10:48633208 | Alt | 0.786, 0.048 | 40.622 | 1.85E-10 |
|  | Intron | chr10:48633218 | Alt | 0.786, 0.048 | 40.622 | 1.85E-10 |
|  | Intron | chr10:48633260 | Alt | 0.786, 0.048 | 40.622 | 1.85E-10 |
|  | Synonymous | chr10:48633309 | Alt | 1.000, 0.929 | 2.09 | 0.1483 |
|  | Nonsynonymous | chr10:48633379 | Alt | 0.786, 0.048 | 40.622 | 1.85E-10 |
|  | Synonymous | chr10:48633417 | Alt | 1.000, 0.952 | 1.373 | 0.2414 |
|  | Intron | chr10:48633436 | Alt | 0.786, 0.048 | 40.622 | 1.85E-10 |
|  | Intron | chr10:48633461 | Alt | 0.786, 0.048 | 40.622 | 1.85E-10 |
|  | Intron | chr10:48633468 | Alt | 0.786, 0.048 | 40.622 | 1.85E-10 |
|  | Intron | chr10:48633485 | Alt | 1.000, 0.810 | 6.022 | 0.0141 |
|  | Intron | chr10:48633508 | Alt | 0.786, 0.048 | 40.622 | 1.85E-10 |
| RYR2 | Intron | chr04:2588820 | Alt | 0.714, 0.238 | 15.556 | 8.01E-05 |
|  | Nonsynonymous | chr04:2589113 | Alt | 0.643, 0.000 | 36.346 | 1.65E-09 |
|  | Synonymous | chr04:2589258 | Alt | 0.679, 0.071 | 28.736 | 8.30E-08 |
|  | Nonsynonymous | chr04:2778722 | Alt | 0.643, 0.000 | 36.346 | 1.65E-09 |
