# Supplementary material for: Hypoxia Adaptations in the Grey Wolf (Canis lupus chanco) from Qinghai-Tibet Plateau
Source: PLoS Genet. 2014 Jul 31;10(7):e1004466. doi: 10.1371/journal.pgen.1004466 (PMC4117439; doi:10.1371/journal.pgen.1004466)
Supplement: Table S10 — Microsatellite loci in this study. (DOC) [file pgen.1004466.s013.doc]

Table S10: Microsatellite loci in this study.

| Primer Name | Sequence (5'---3') | Primer Name | Sequence (5'---3') |
| --- | --- | --- | --- |
| FH2001F | TCCTCCTCTTCTTTCCATTGG | FH2869F | CATTAACAAAATTGCTTCAGTCC |
| FH2001R | TGAACAGAGTTAAGGATAGACACG | FH2869R | CACACCAATAACCCAGAACG |
| FH2004F | CTAAGTGGGGAGCCTCCTCT | FH2914F | GTGATCCACTTGCTTGTATCC |
| FH2004R | ACTGTGACCTACTGAGGTTGCA | FH2914R | ATAGCCTTGGGAATTTTTGC |
| FH2010F | AAATGGAACAGTTGAGCATGC | FH3047F | GCCAAGATATGGAAACAACC |
| FH2010R | CCCCTTACAGCTTCATTTTCC | FH3047R | ATCAGATAGCTGGGTGATGG |
| FH2054F | GCCTTATTCATTGCAGTTAGGG | FH3398F | CAAAGTTCAAACAGGAATGG |
| FH2054R | ATGCTGAGTTTTGAACTTTCC | FH3398R | GTTCTGTGCTGAGCATTGG |
| FH2088F | CCCTCTGCCTACATCTCTGC | FH3399F | TCTCTATGCCTGCAGTTTCC |
| FH2088R | TAGGGCATGCATATAACCAGC | FH3399R | TTCTGATGCCCTCATAAAGC |
| FH2137F | GCAGTCCCTTATTCCAACATG | FH3725F | GAAAGAACTCACTCAAAACTTCC |
| FH2137R | CCCCAAGTTTTGCATCTGTT | FH3725R | AAATGTTACTTCAGAAAAGCTGG |
| FH2324F | AGCTCTATGAAAGGTGATTGCC | FH3853F | ATAGCCAAAAGGTAGAAATAATCC |
| FH2324R | AGACAGCCATACAAATGAGAATTG | FH3853R | GTAAGAGGGAGCACAAGTGG |
| FH2611F | GAAGCCTATGAGCCAGATCA | FH3965F | GTCGCTCAGCAGTTAAGCTC |
| FH2611R | TGTTAGATGATGCCTTCCTTCT | FH3965R | GAATCCTGGCTCTGCTACTTAC |
| FH2658F | TCTTAGAAATTGCTGGTGGG | PEZ5F | GCTATCTTGTTTCCCACAGC |
| FH2658R | TAAGAAACTGCCAGTCTGTGG | PEZ5R | TCACTGTATACAACATTGTC |
| FH2670F | GTGGAGTCCTGTGTCTTGATG | PEZ8F | TATCGACTTTATCACTGTGG |
| FH2670R | CTTCTGTTGAAAAATGCAGTCA | PEZ8R | ATGGAGCCTCATGTCTCATC |
| FH2766F | TTTCAAGAAAGGCAGAAAGTG | PEZ12F | GTAGATTAGATCTCAGGCAG |
| FH2766R | TTAAGGGAGCCATGAATCTG | PEZ12R | TAGGTCCTGGTAGGGTGTGG |
| FH2785F | ATGGCAGGTCAAGAGTATGG | PEZ15F | CTGGGGCTTAACTCCAAGTTC |
| FH2785R | GATAGATCCAAGCCAACACC | PEZ15R | CAGTACAGAGTCTGCTTATC |
| FH2790F | CCAATATTGTTAAGAAGTTCAAGC | PEZ19F | GACTCATGATGTTGTGTATC |
| FH2790R | AGGCCTTCTCTGTCCTCTTG | PEZ19R | TTTGCTCAGTGCTAAGTCTC |
