# Supplementary material for: Hypoxia Adaptations in the Grey Wolf (Canis lupus chanco) from Qinghai-Tibet Plateau
Source: PLoS Genet. 2014 Jul 31;10(7):e1004466. doi: 10.1371/journal.pgen.1004466 (PMC4117439; doi:10.1371/journal.pgen.1004466)
Supplement: Table S12 — Transitions: transversions ratio (Ti:Tv) for each individual using sites pass GF2 and SF filters. (DOC) [file pgen.1004466.s015.doc]

Table S12 Transitions: transversions ratio for each individual using sites pass GF2 and SF filters.

| **Samples** | **Transitions/transversions ratio** |
| --- | --- |
| RKWL | 2.341 |
| IM06 | 2.329 |
| IM07 | 2.321 |
| QH11 | 2.333 |
| QH16 | 2.339 |
| TI09 | 2.355 |
| TI32 | 2.353 |
| XJ24 | 2.349 |
| XJ30 | 2.347 |
