# Supplementary material for: Hypoxia Adaptations in the Grey Wolf (Canis lupus chanco) from Qinghai-Tibet Plateau
Source: PLoS Genet. 2014 Jul 31;10(7):e1004466. doi: 10.1371/journal.pgen.1004466 (PMC4117439; doi:10.1371/journal.pgen.1004466)
Supplement: Protocol S1 — Genotyping pipeline. (DOCX) [file pgen.1004466.s016.docx]

**Genotyping pipeline**

After mapping the short reads to the reference genome, we applied two major tools, Picard ([http://picard.sourceforge.ne](http://picard.sourceforge.net)t) and Genome Analysis Toolkit (GATK) toolset [1], to process the alignments to genotype calls. The whole pipeline converted the short reads to bam format alignment files [2], and then got the genotype calls as in vcf format (http://www.1000genomes.org/node/101) from bam files after multiple steps (Figure S3). We described the details of our pipeline in here.

**S1. Local realignment**

The false SNPs could be detected in regions where repeated alignment errors occur across overlapping reads because short read alignment algorithms work on each read independently. In order to reduce the false positive SNPs, we used GATK IndelRealigner [1] to perform local multiple alignment. In total, there were three steps. First, identify suspicious intervals that may require realignment, then performing local realignment within these intervals, and then fix the mate pairing lost during the local realignment process. Example command lines were:

**a. Interval detection**

java -Xmx9g -jar GenomeAnalysisTK.jar -T RealignerTargetCreator --read_filter BadCigar -R reference_genome.fa -I input_file.bam -o output.intervals

**b. Local realignment**

java -Xmx9g –jar GenomeAnalysisTK.jar -T IndelRealigner –R reference_genome.fa -I input_file.bam -targetIntervals output.intervals -o output_realign.bam

**c. Fix the mate pair information**

java -jar -Xmx9g FixMateInformation.jar INPUT=output_realign.bam OUTPUT=output_realign_fixed.bam SORT_ORDER=coordinate VALIDATION_STRINGENCY=LENIENT

**S2. Base quality recalibration**

Quality scores assigned to individual base calls only reflected confidence in the specified nucleotide, but they were may be weakly correlated with the actual probabilities of erroneous base calls [Freedman et al. Plos Genet, In review]. Therefore, it is necessary to standardize quality scores across sequencing runs and libraries. Here, we performed empirical quality score recalibration using GATK. Three steps were involved: 1) since there was no dbSNP data set for wolf, we genotype in the same way as below (see S3) to liberally define a SNP dataset that are excluded in the subsequent steps; 2) for the rest sites, creating the table for the frequency of base calls that are correct v.s. incorrect as a function of covariates reflecting features of the underlying sequence context stratified by library/sequencing run; 3) using the genome-wide empirical error rates conditional on each unique covariate set to replace the original quality scores. Example command lines were:

**a. Create recalibration table**

java -jar GenomeAnalysisTK.jar -R reference_genome.fa -T CountCovariates -l INFO -cov ReadGroupCovariate -cov CycleCovariate -cov DinucCovariate -cov QualityScoreCovariate --default_platform Illumina -I input_file.bam --knownSites:VCF Recalibration_Input.vcf -recalFile output_retable.csv --solid_recal_mode SET_Q_ZERO --solid_nocall_strategy LEAVE_READ_UNRECALIBRATED

**b. Generate recalibrated bam files**

java -jar -Xmx9g GenomeAnalysisTK.jar -R reference_genome.fa -l INFO -T TableRecalibration --default_platform Illumina -I input_file.bam -o Recal_output.bam -recalFile output_retable.csv --doNotWriteOriginalQuals --solid_recal_mode SET_Q_ZERO --solid_nocall_strategy PURGE_READ

**S3. SNP and Indel calling**

We used the GATK Unified Genotyper [1] to call genotypes for all the samples. Because several different conservative post-genotyping filters were applied later, we set both standard minimum confidence thresholds to zero here. Example command line was:

java -jar -Xmx10g GenomeAnalysisTK.jar -R reference_genome.fa -T UnifiedGenotyper

-l INFO --genotyping_mode DISCOVERY --output_mode EMIT_ALL_CONFIDENT_SITES -I input_file.bam --min_base_quality_score 20 --standard_min_confidence_threshold_for_emitting 0.0 --standard_min_confidence_threshold_for_calling 0.0 -A GCContent -o output.vcf -metrics output.metrics -dt NONE

Accurate indel calling from short reads is still subject to considerable [Freedman et al. Plos Genet, In review], and there is little prior information about the distribution of indels in wolf genome. Furthermore, we did not have method to validate indel calls at the genome-wide scale in a manner comparable to that available for SNP calls. Thus, we called indels only for use in the filtering out of SNPs proximate to them that might be false positives, accepting that the indel calls are only approximations. Example command line was:

java -jar -Xmx8g GenomeAnalysisTK.jar -R reference_genome.fa -T UnifiedGenotyper -l INFO --genotyping_mode DISCOVERY --output_mode EMIT_ALL_CONFIDENT_SITES -I input_file.bam -glm INDEL --indel_heterozygosity 0.000125 --min_indel_count_for_genotyping 5 --min_base_quality_score 20 --standard_min_confidence_threshold_for_emitting 0.0 --standard_min_confidence_threshold_for_calling 0.0 -A GCContent -o output.vcf -metrics output.metrics -dt NONE

**Reference:**

1. DePristo MA, Banks E, Poplin R, Garimella KV, Maguire JR**,** et al. (2011) A framework for variation discovery and genotyping using next-generation DNA sequencing data. Nat Genet 43: 491-498.

2. Li H, Handsaker B, Wysoker A, Fennell T, Ruan J, et al. (2009) The Sequence Alignment/Map format and SAMtools. Bioinformatics 25: 2078-2079.
