# Supplementary material for: Hypoxia Adaptations in the Grey Wolf (Canis lupus chanco) from Qinghai-Tibet Plateau
Source: PLoS Genet. 2014 Jul 31;10(7):e1004466. doi: 10.1371/journal.pgen.1004466 (PMC4117439; doi:10.1371/journal.pgen.1004466)
Supplement: Protocol S2 — Post genotype filters. (DOCX) [file pgen.1004466.s017.docx]

**S4 Post-genotype filters**

After having the genotype calls from GATK, we applied several conservative data quality filters to control the data quality again. There were two levels of filters: genome filters (GF, which is based on the reference genome’s features and polymorphism across samples) and sample filters (SF, which is based on the genotype calls of each sample). We described the details of the filters below.

**S4.1 Genome filters**

***S4.1.1. Triallelic sites (MA).*** Triallelic sites were more prone to genotyping errors [Freeman et al., Plos Genet, In review]. Besides, such site only contains a small fraction of the genome (0.002%). Thus we filter out all the triallelic sites.

***S4.1.2. Copy Number Variants (CNV).*** Misalignment is possible when short reads mapped to the places of reference where contain novel CNVs. It can lead to false positive SNPs. To minimize this type of misalignment, we applied a set of CNV regions to filter out from downstream analyses. Since we did not detect CNVs in this study, we used previously discovered CNVs reported in reference genome and in a diverse panel of dog breeds [1-2, Freeman et al., Plos Genet, In review].

***S4.1.3. Repeat Regions (RR).*** The repeat regions of the reference genome were identified with RepeatMasker [3] and Tandem Repeat Finder [4]. A large portion of the genome was repeat regions, but ancient repeats have diverged enough to allow accurate read mapping with short read alignment algorithms [Freeman et al., Plos Genet, In review], thus we only filtered out younger repeats prone to sequence misalignment. Freedman et al [Plos Genet, In Review] used 25% divergence as minimum repeat divergence threshold in six canids because they found that repeats in the ancient repeats show no increase in heterozygosity with decreasing repeat age.

***S4.1.4. CpG.*** Mutation rate at CpG sites is higher than non-CpG sites [5], so that regions enriched for CpGs may display elevated diversity and/or divergence leading to outliers in window-based analyses. We flagged any sites that even one of the samples fell within a CpG dinucleotide.

**S4.2 Sample filters**

***S4.2.1. Proximity to Indel (DL).*** Short reads are prone to misalignment near indels [Freeman et al., Plos Genet, In review], and the local realignment around indels in our genotyping pipeline may not fully fix this problem. Therefore, to minimize the potential source of bias, for each sample we excluded any SNPs near indels (5bp, either up or downstream).

***S4.2.2. Genotype Quality (GQ).*** Genotype quality is the phred-scaled probabilities (10*log10(P[error]).), which represent the genotype calls do not match the true genotype. Hard genotype quality thresholds work well with high coverage (>20x), although it may cause underestimate of heterozygotes in low or moderate coverage genomes [6]. All the samples in our study were sequenced at >20x. Moreover, the distribution of genotype quality (Figure S4.1) showed that large proportion of SNP sites have GQ > 20 (IM06: 95.21%; IM07: 94.15%; XJ24: 94.98%; XJ30: 95.62%; QH11: 95.68%; QH16: 94.52%; TI09: 93.97%; TI32: 93.69%; RKWL: 95.80%). Therefore, we chose a hard minimum GQ threshold of 20 (P[error]=0.01).

***S4.2.3. Depth of Coverage (DP).***

***S4.2.3.1. Excess Depth of Coverage for all sites.*** Extremely high depth of coverage relative to the genome-wide average likely indicates misalignment of reads generated from paralogous positions in the genome. Indeed, excess depth of coverage is a typical metric used to define CNV regions, but CNV filtering alone will fail to detect finer-resolution CNV signatures [Freeman et al., Plos Genet, In review]. Therefore, we conservatively filtered all sites if their depth of coverage exceeded twice the mean depth of coverage of each sample.

***S4.2.3.2. Minimum Depth of Coverage for non-variant sites.*** Since only the very old version of GATK will output the GQ value for non-variant sites and the version we are using does not, thus we did not have GQ filters for non-variant sites. Instead, we used minimum depth of coverage as one of the filters for non-variant sites. Here, we set the minimum threshold as eight.

***S4.2.4. Clustered SNPs (DV).*** Within any sample, we excluded all SNPs that within 5 bp of another SNP.

**S4.3 Combination of filters**

For different types of analyses, we used different combination of GF and SF filters. GF1 and SF were used to analyze involving estimation of genome-wide patterns of diversity; GF2 and SF were used to analyze functional regions. The combination of the filters was:

**S4.3.1. Non-variant sites:**

GF1: CNV, CpG, RR

GF2: CNV, RR

SF: DP >= 8, DP <= (2 x mean coverage)

**S4.3.2. SNP sites:**

GF1: MA, CNV, CpG, RR

GF2: MA, CNV, RR

SF: GQ >= 20, DP <= (2 x mean coverage), DL, DV

Figure S4.1 Genomic coverage per sample as a function of genotype quality before adding any other filters. Numbers in legend were mean genome-wide coverage.

**Reference**

1. Nicholas TJ, Baker C, Eichler EE, Akey JM. (2011) A high-resolution integrated map of copy number polymorphisms within and between breeds of the modern domesticated dog. BMC Genomics 12:414.

2. Axelsson E, Ratnakumar A, Arendt ML, et al. (2013) The genomic signature of dog domestication reveals adaptation to a starch-rich diet. Nature 495: 360-364.

3. Smit AFA, Hubley R, Green P. (1996-2010) RepeatMasker Open-3.0.

4. Benson G (1999) Tandem repeats finder: a program to analyze DNA sequences. Nucleic Acids Res 27: 573-580.

5. Hodgkinson A, Eyre-Walker A (2011) Variation in the mutation rate across mammalian genomes. Nat Rev Genet 12: 756-766.

6. Nielsen R, Paul JS, Albrechtsen A, Song YS (2011) Genotype and SNP calling from next-generation sequencing data. Nat Rev Genet 12: 443-451.
